# Decoupling Dopamine Synthesis from Impulsive Action, Risk-Related Decision-Making, and Propensity to Cocaine Intake: A Longitudinal [^18^F]-FDOPA PET Study in Roman High- and Low-Avoidance Rats

**DOI:** 10.1523/ENEURO.0492-23.2023

**Published:** 2024-02-09

**Authors:** Ginna Urueña-Méndez, Chloé Arrondeau, Lidia Bellés, Nathalie Ginovart

**Affiliations:** ^1^Departments of Psychiatry, Faculty of Medicine, University of Geneva, Geneva CH1206, Switzerland; ^2^Basic Neuroscience, Faculty of Medicine, University of Geneva, Geneva CH1206, Switzerland

**Keywords:** [^18^F]-FDOPA, cocaine SA, DA synthesis, impulsive action, risk-related decision-making

## Abstract

Impulsive action and risk-related decision-making (RDM) are two facets of impulsivity linked to a hyperdopaminergic release in the striatum and an increased propensity to cocaine intake. We previously showed that with repeated cocaine exposure, this initial hyperdopaminergic release is blunted in impulsive animals, potentially signaling drug-induced tolerance. Whether such dopaminergic dynamics involve changes in dopamine (DA) synthesis as a function of impulsivity is currently unknown. Here, we investigated the predictive value of DA synthesis for impulsive action, RDM, and the propensity to take cocaine in a rat model of vulnerability to cocaine abuse. Additionally, we assessed the effects of cocaine intake on these variables. Rats were tested sequentially in the rat Gambling Task (rGT) and were scanned with positron emission tomography and [^18^F]-FDOPA to respectively assess both impulsivity facets and striatal DA synthesis before and after cocaine self-administration (SA). Our results revealed that baseline striatal levels of DA synthesis did not significantly predict impulsive action, RDM, or a greater propensity to cocaine SA in impulsive animals. Besides, we showed that impulsive action, but not RDM, predicted higher rates of cocaine taking. However, chronic cocaine exposure had no impact on DA synthesis, nor affected impulsive action and RDM. These findings indicate that the hyper-responsive DA system associated with impulsivity and a propensity for cocaine consumption, along with the reduction in this hyper-responsive DA state in impulsive animals with a history of cocaine use, might not be mediated by dynamic changes in DA synthesis.

## Significance Statement

Impulsive behaviors are associated with a heightened presynaptic dopamine (DA) function and vulnerability to the rewarding effects of cocaine. However, with repeated drug exposure, the initially high DA release decreases, probably reflecting the development of drug tolerance. Whether such DA dynamics involve changes in DA synthesis is currently unknown. Using in vivo neuroimaging in rats before and after chronic cocaine use, our study reveals that DA synthesis does not predict impulsivity or vulnerability to cocaine, nor is it affected by chronic drug exposure. Our results suggest that the heightened presynaptic function underlying impulsivity and the cocaine-induced tolerance to drugs depend on alternative mechanisms to DA synthesis, such as those controlling DA reactivity to stimulation and DA reuptake.

## Introduction

Impulsive action and risk-related decision-making (RDM) are two facets of impulsivity differentially linked to individual vulnerability to cocaine abuse (Dalley and Robbins, 2017). While impulsive action predicts greater cocaine intake (Dalley et al., 2007; Arrondeau et al., 2023), even under punishment (Belin et al., 2008), RDM predicts the incubation of craving (Ferland and Winstanley, 2017). Despite their differential contribution to the risk of cocaine abuse, both impulsivity facets share dopaminergic alterations akin to those observed in drug abusers (Dalley and Robbins, 2017). For instance, humans with high trait impulsivity exhibit reductions in striatal dopamine (DA) D_2/3_ receptors (D_2/3_R; Caravaggio et al., 2016) as those observed in cocaine abusers (Volkow et al., 1993; Ashok et al., 2017). Such reduction in striatal D_2/3_R also occurs in rodents with high impulsive action (Dalley et al., 2007; Bellés et al., 2021) and RDM (Simon et al., 2011) and predicts a higher cocaine intake (Dalley et al., 2007; Bellés et al., 2021). Additionally, trait impulsivity and RDM are linked to heightened DA release in response to psychostimulants in humans (Buckholtz et al., 2010; Oswald et al., 2015), and such elevated DA release has also been confirmed in impulsive rats (Bellés et al., 2021). Importantly, heightened DA release predicts striatal D_2/3_R deficits, suggesting that those deficits might represent a compensation for heightened DA levels in impulsive animals (Bellés et al., 2021). Moreover, hyperdopaminergic release in impulsive animals predicts a higher propensity to cocaine self-administration (SA; Urueña-Mendez et al., 2023). Together, these studies strengthen the view that increased presynaptic—rather than decreased postsynaptic—DA function might be the main dysregulation underlying impulsive behaviors, conferring greater vulnerability to cocaine abuse.

Although the molecular underpinnings of such heightened DA function in impulsive subjects are unknown, one potential underlying mechanism could be increased DA synthesis. Indeed, tyrosine hydroxylase (TH) expression is higher in the midbrain of innately high-impulsive rats relative to low-impulsive rats (Tournier et al., 2013). However, no study has yet evaluated whether impulsive action and RDM are associated with alterations in DA synthesis and how such alterations might predict the propensity to take cocaine.

Besides the predictive value of impulsivity facets and DA function in cocaine abuse, there is also evidence that chronic cocaine exposure can affect impulsive behaviors and DA signaling (Dalley and Robbins, 2017). However, cocaine effects may vary depending on the impulsivity facet assessed. While several studies (Dalley et al., 2005; Ferland and Winstanley, 2017; Abbott et al., 2022; Urueña-Mendez et al., 2023) have reported no alterations in impulsive action shortly after cocaine withdrawal (but see Dalley et al., 2007; Winstanley et al., 2009), research on RDM is scarce and has yielded mixed results. For instance, one study (Ferland and Winstanley, 2017) found impaired RDM exclusively in risky rats (i.e., those preferring high reward magnitude at higher punishment risk), while another study found impairments in nonrisky rats (Cocker et al., 2019). Such inconsistencies highlight the need for further longitudinal studies to clarify the consequences of chronic cocaine use on different impulsivity facets. Chronic cocaine use also leads to neuroadaptive alterations in DA signaling. Indeed, chronic cocaine exposure attenuates DA release in response to psychostimulants in impulsive rats (Urueña-Mendez et al., 2023), which is consistent with the blunted DA release observed in cocaine abusers (Martinez et al., 2007; Volkow et al., 2014). Although the mechanisms underlying such dopaminergic tolerance to psychostimulants are unclear, one study on cocaine abusers suggests that it could involve reductions in DA synthesis (Wu et al., 1997). However, the study's results have been debated (Cumming et al., 1999), and no additional research on cocaine abusers has been performed to confirm this finding. Similarly, no preclinical studies have ever assessed whether chronic cocaine use affects DA synthesis in relation to impulsivity.

Altogether, current research links impulsive action and RDM to greater DA release and higher risk of cocaine abuse and also indicates that chronic cocaine use can induce DA tolerance to psychostimulants. However, whether such DA dynamics involve changes in DA synthesis is currently unknown.

Here, we used in vivo positron emission tomography (PET) and the L-DOPA radiolabelled analog ^18^F-fluoro-dihydroxyphenylalanine ([^18^F]-FDOPA) to investigate the predictive value of striatal DA synthesis on impulsive action, RDM, and the propensity to cocaine SA in the Roman high- (RHA) and low-avoidance (RLA) rat model of vulnerability to drug abuse (Giorgi et al., 2019). Additionally, we evaluated the effects of chronic cocaine intake on impulsive behaviors and DA synthesis. Our results shed light on the DA mechanisms underlying impulsivity and vulnerability to the rewarding effects of cocaine.

## Materials and Methods

### Subjects

Subjects were outbred male rats RLA (*n* = 22) and RHA (*n* = 23) from our permanent colony at the University of Geneva. Rats were paired or trio-housed in a temperature-controlled room (22 ± 2°C) under a 12 h light/dark cycle (lights on at 07:00 A.M.). Food was restricted daily (5 g per 100 g of body weight) throughout the experiment, and water was provided ad libitum. All experiments were approved by the animal ethics committee of the Canton of Geneva and performed according to the Swiss federal law on animal care.

### General procedure

**Figure 1.**
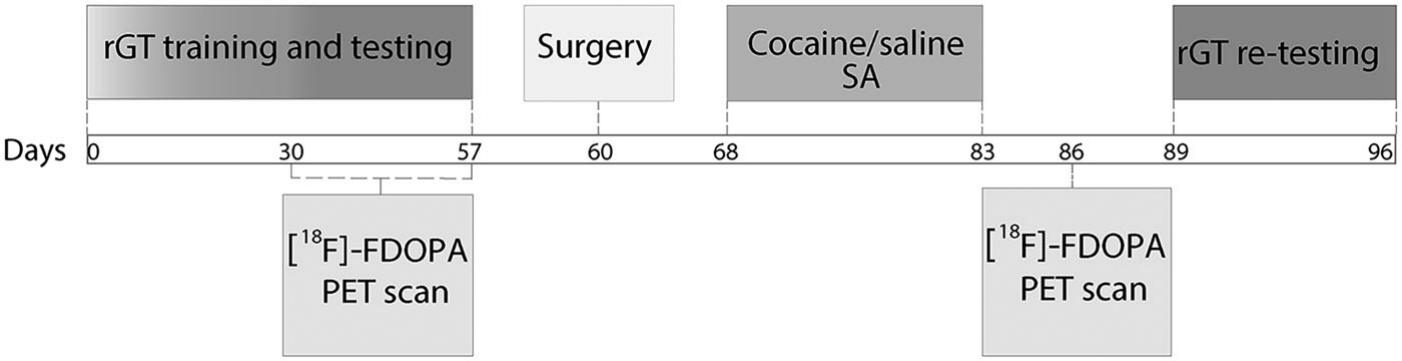
Timeline of the study with specific time points (in days) annotated in the main line.

Figure 1 shows the timeline of the study. Two cohorts of rats (*n* = 22–23 per line) were first trained and tested in the rat Gambling Task (rGT) to concurrently measure their baseline levels of impulsive action and RDM. Next, a subgroup of rats in each cohort (16 rats per line) was scanned with PET and [^18^F]-FDOPA to assess their baseline capacity to synthesize DA. Rats were then trained to self-administer either cocaine (*n* = 14–15 rats per line) or saline vehicle (*n* = 8 rats per line). Three days after the last SA session, the subgroup of rats scanned at baseline received a second [^18^F]-FDOPA PET scan. Finally, starting at day 6 post-SA, all rats were retested in the rGT for 8 d. This procedure allows us to investigate the effects of repeated cocaine exposure on DA synthesis capacity, impulsive action, and RDM.

### Rat Gambling Task

The rGT task was performed in 11 standard operant conditioning chambers (Med Associates). Each chamber was fitted with a house light and a food magazine. On the opposite wall, five response holes (the central hole being inactivated during the task) were fitted with a cue light and infrared beams to detect nose-poke responses.

Testing occurred daily (5 d/week) in 30 min sessions performed between 8 and 12 h following a protocol previously described (Zeeb et al., 2009). In brief, animals learned to nose-poke in one illuminated (i.e., active) hole to obtain a food pellet (purified rodent tablets of 45 mg, TestDiet, Sandown Scientific). Subsequently, rGT options were introduced over seven forced-choice sessions. The options were termed P1, P2, P3, and P4. They varied in the number of pellets delivered (1, 2, 3, or 4, respectively), the probability of pellet delivery (0.9, 0.8, 0.5, or 0.4, respectively), the time-out punishment duration (5 s, 10 s, 30 s, or 40 s, respectively), and the time-out punishment probability (0.1, 0.2, 0.5, or 0.6, respectively) for the nonrewarded trials. The options were associated with specific holes counterbalanced between animals.

Rats initiated each trial by nose-poking into the food magazine. This response triggered a 5 s intertrial interval (ITI). Once the ITI had elapsed, one option was pseudorandomly presented (i.e., the corresponding hole lit), and the rat had 10 s to nose-poke. This response was followed by reward or punishment according to the hole-associated contingency. During punished trials, the hole light flashed at 0.5 Hz. After completing the forced-choice training, rats performed the rGT task (i.e., with all the options presented simultaneously) during 25 sessions to establish a stable response at baseline. A choice score [(%P1+%P2)–(%P3+%P4)] was used as an index of RDM (Zeeb and Winstanley, 2013; Fugariu et al., 2020; Hultman et al., 2022), with a lower choice score indicating less optimal decision-making. Impulsive action was defined as the percentage of premature responses performed during the ITI (i.e., number of premature responses/total number of trials initiated × 100; Ferland and Winstanley, 2017). Both outcome measures were calculated at baseline and after cocaine SA as the average across three consecutive sessions of stable performance, with no main effect of session and no session-by-choice score interaction as assessed using a repeated-measures analysis of variance (ANOVA).

### [^18^F]-FDOPA PET imaging

Animals were fasted for 12 h before the PET scan. To prevent early breakdown and decarboxylation of [^18^F]-FDOPA (Firnau et al., 1986; Sawle et al., 1994), rats received 40 mg/kg (i.p.) of entacapone (Combi-Blocks) 1 h before the scan and 10 mg/kg (i.p.) benserazide hydrochloride (Sigma-Aldrich) 30 min before the scan, respectively. Under isoflurane anesthesia, two animals per scan were positioned in the micro-PET scanner Triumph II (TriFoil Imaging) using a custom-made stereotactic-like frame. After a 5 min computer tomography (CT) scan, rats received an intravenous bolus injection of 58 ± 9 MBq of [^18^F]-FDOPA (Advanced Accelerator Applications) and were scanned for 60 min.

Dynamic PET images were acquired in list mode and reconstructed into 19 timeframes (2 × 30 s, 2 × 60 s, 4 × 120 s, 3 × 180 s, 8 × 300 s) using the ordered subsets expectation maximization (OSEM) algorithm with 30 iterations. Image analysis was conducted with PMOD software (version 4.0, PMOD Technologies Ltd., Zurich, Switzerland). PET summation images were generated from dynamic PET data and checked for alignment with the corresponding CT images. Because minor motion occurred between the PET and CT images, all PET images were coregistered to the corresponding CT images using rigid registration based on the normalized mutual information. CT images were then coregistered to a magnetic resonance image (MRI) brain template of the rat brain (Schiffer et al., 2006) using mutual information-based rigid body registration. The resulting transformation was then applied to the corresponding PET dynamic images, mapping all rat brains into the same reference space.

A region of interest (ROI) template was defined on the MRI atlas of the rat brain (Schweinhardt et al., 2003), as previously described (Ginovart et al., 2012). This template included three brain regions: the dorsolateral striatum (DST), the ventral striatum (VST), and the cerebellar cortex. ROIs (2 mm diameter circles) for the DST and VST were placed on the central planes of each structure to minimize the partial volume effect. A single elliptical ROI (2 mm × 1 mm) was placed on the cerebellar cortex, primarily over the central lobule, to reduce spillover from bone uptake of [^18^F]-fluoride. The ROI template was applied to the dynamic images to produce time-activity curves for the target-rich (DST) and reference region (cerebellar cortex), which were then used to generate parametric maps. Voxel-wise parametric maps of the [^18^F]-FDOPA influx rate constant (*k_i_*^cer^, min^−1^) were calculated using the Patlak multiple-time graphical analysis (Patlak and Blasberg, 1985) using the cerebellum as a reference to estimate the free and nonspecifically bound radiotracer. The ROI template was then applied to the individual parametric maps to extract regional *k_i_*^cer^ estimates as an index of DA synthesis. Both hemisphere data were averaged to obtain a single *k_i_*^cer^ value for the DST and VST.

### Cocaine self-administration

The SA procedure was performed as described in Dimiziani et al. (2019). In brief, rats were implanted with a polyurethane catheter (Instech Laboratories) into the right jugular vein under 2.0% isoflurane anesthesia. The catheter was fixed in the midscapular region and attached to a vascular access button (VAB) protected from cage mate chewing with an aluminum cap (Instech Laboratories). Rats received postoperatory care and were allowed to recover for 1 week before SA training. The catheter's patency was maintained by infusing 0.1 ml of heparinized saline (30 U.I./ml) daily.

Training occurred in 11 standard operant conditioning chambers (Med Associates) equipped with a house light and two response holes connected to an external infusion pump. The holes were fitted with a cue light and infrared beams to detect nose-poke responses. Rats were trained to self-administer 0.3 mg/kg/infusion of cocaine hydrochloride (Geneva, University Hospitals) or 0.9% saline vehicle under a fixed ratio 1 schedule (FR1). Sessions were performed daily (5 d/week) for 3 weeks and lasted 2 h or until the animals reached the maximum 60 infusions allowed. Each session started with the illumination of the house light and a priming infusion of the assigned treatment (cocaine or saline). Nose-poke responses in a specific hole (active) extinguished the house light and turned on the hole-cue light while delivering one infusion of 0.06–0.1 ml of cocaine or saline over 2.5–4 s. At the end of each infusion, the hole-cue light extinguished, signaling the beginning of a 20 s time-out (TO). Nose-poke responses during the TO had no programmed consequences. Responses in the other hole (inactive) were recorded but delivered no infusions. The location of the active and inactive holes was randomly counterbalanced between animals.

Cocaine SA acquisition was defined as the obtention of ≥15 infusions during 2 consecutive days with accurate discrimination (70%) of the active versus inactive hole. Cocaine maintenance was defined as a stable drug intake (≤15% variation in the number of infusions) over 3 consecutive days. We evaluated the total number of infusions and rate of infusions as measures of drug intake and the total nose-poke responses in the active versus inactive hole as a measure of hole discrimination/preference. For correlation analysis, the three last SA sessions were averaged to obtain a single value for each variable.

### Statistical analysis

All statistical analyses were conducted with SPSS Statistics 26.0 (IBM) software, except the Grubbs’ test (at *p* < 0.01) for outlier detection performed in GraphPad Prism 9.0 (GraphPad Software 9.0). Normality of data distribution was verified using the Shapiro–Wilk statistic at *p* < 0.05, and non-normal data were LOG-10 transformed before the ANOVA analysis, as specified below. All ANOVA analyses were followed by Bonferroni’s post hoc tests when appropriate.

For baseline comparisons, rats were grouped by line (RHA or RLA), irrespective of their subsequent treatment (saline or cocaine) assignment. Between-line differences in the percentage of premature responses and choice score were analyzed with an unpaired *t* test or Mann–Whitney *U* statistic, depending on the data distribution. Baseline differences in [^18^F]-FDOPA *k_i_*^cer^ values were assessed using a two-way repeated-measures ANOVA with line as between-subjects factor and brain region (DST or VST) as within-subjects factor.

The percentage of animals reaching the criteria for cocaine SA acquisition was evaluated using the Kaplan–Meier survival analysis. The discrimination of the active versus inactive hole during cocaine acquisition and maintenance was evaluated using a mixed factorial ANOVA, with session (days 1–15) and hole (active or inactive) as within-subjects factors and with line and treatment as between-subjects factors. In the measure of total nose-poke responses in the active hole, Grubbs’ test identified one RLA outlier within the cocaine group (*G* = 3.3; *p* < 0.01). We applied the Winsorize method to correct for this outlier value in the ANOVA analysis.

The number of infusions and the rate of infusions displayed during acquisition and maintenance of cocaine SA were analyzed using a mixed factorial ANOVA, with session as within-subjects factor, and line and treatment as a between-subjects factors.

The effect of repeated cocaine exposure on premature responding, choice score, and [^18^F]-FDOPA *k_i_*^cer^ was evaluated using a mixed factorial ANOVA, with time (i.e., pre- or post-SA) as within-subjects factor and line and treatment as between-subjects factors. Due to a technical failure in [^18^F]-FDOPA radiopharmaceutical synthesis, 1 RHA and 2 RLA rats did not receive their PET scan following SA and were therefore not included in the analysis.

Finally, correlations between [^18^F]-FDOPA *k_i_*^cer^, rGT, and SA variables were tested using Pearson's or Spearman's correlation according to the data distribution.

## Results

### Baseline impulsive action and risk-related risky decision-making

**Figure 2.**
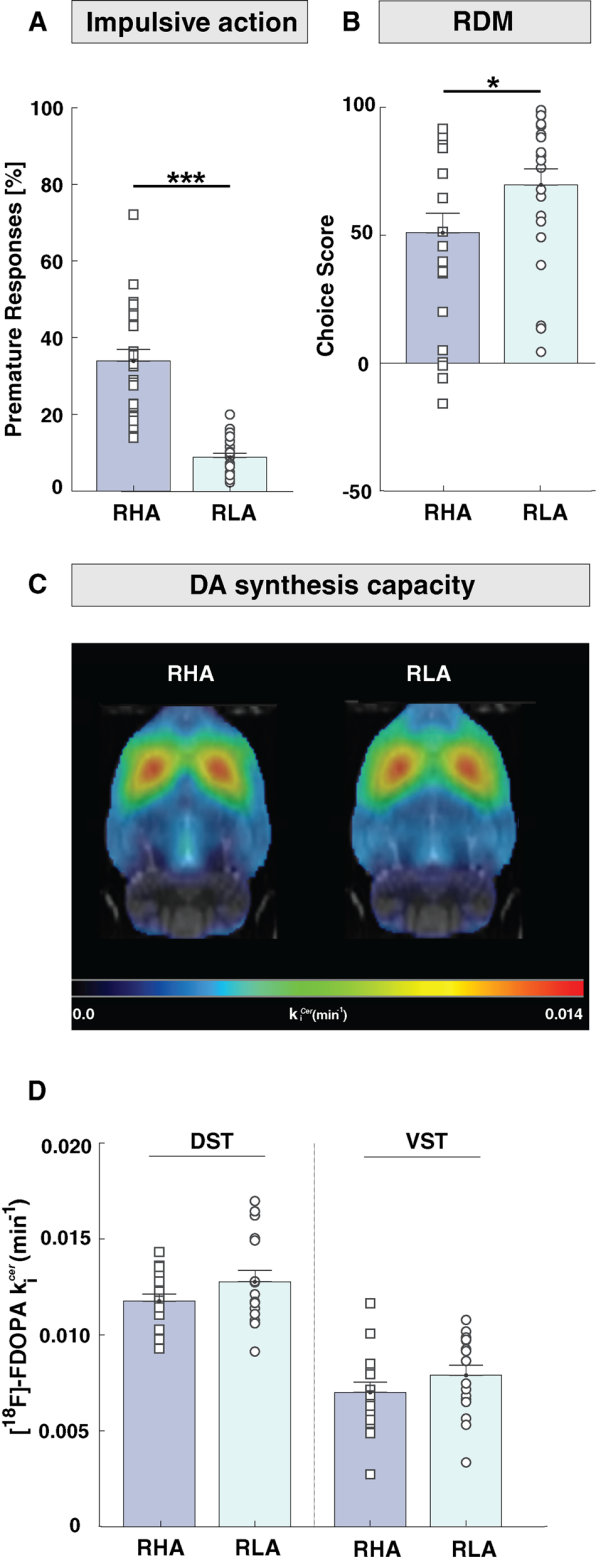
Baseline measures of impulsive action, RDM, and striatal DA synthesis capacity in RHA and RLA rats. ***A***, The percentage of premature responses, as an index of impulsive action, was significantly higher in RHAs compared with RLAs. ***B***, Choice score, as an index of RDM, was significantly lower in RHAs compared with that in RLAs. ***C***, Mean parametric maps of [^18^F]-FDOPA *k_i_*^cer^ in RHA and RLA rats. *k_i_*^cer^ parametric maps are projected upon the MRI rat atlas (grayscale) and are shown in horizontal planes at the level of the DST. ***D***, [^18^F]-FDOPA *k_i_*^cer^ did not significantly differ between RHA and RLA rats in either DST or VST. Data are shown as mean ± SEM. Significantly different at *p < 0.05 and ***p < 0.001 in RHAs versus RLAs.

At baseline, RHA rats reached a significantly higher percentage of premature responses relative to RLAs (*t* = 7.7; df = 43; *p* < 0.001; *d* = 2.3; Fig. 2*A*), indicating that RHAs displayed higher levels of impulsive action than RLAs. When evaluating RDM, RHAs displayed significantly lower choice scores than RLAs (*U* = 166; *p* < 0.05; *η*^2^ = 0.14; Fig. 2*B*), indicating less optimal decision-making than RLAs. No difference occurred in the total number of trials initiated by each rat line (*t* = −0.7; df = 43; *p* = 0.49; *d* = 0.20), thus showing that the between-line difference in choice score and premature responding did not result from differences in the trials performed by the two rat lines in the rGT.

### Baseline striatal DA synthesis and impulsive behaviors

Figure 2*C* shows the mean parametric maps for [^18^F]-FDOPA *k_i_*^cer^ obtained in RHA and RLA rats at baseline conditions. In both rat lines, [^18^F]-FDOPA *k_i_*^cer^ values were higher in the DST than those in the VST (region: *F*_(1,30) _= 150.9; *p* < 0.001; *ηp*^2^ = 0.83; Fig. 2*D*). However, we found no significant differences in [^18^F]-FDOPA *k_i_*^cer^ between the two rat lines (line: *F*_(1,30) _= 2.38; *p* = 0.13; *ηp*^2^ = 0.06) in either striatal subdivision (line × region: *F*_(1,30) _= 0.18; *p* = 0.89; *ηp*^2^ = 0.001).

**Figure 3.**
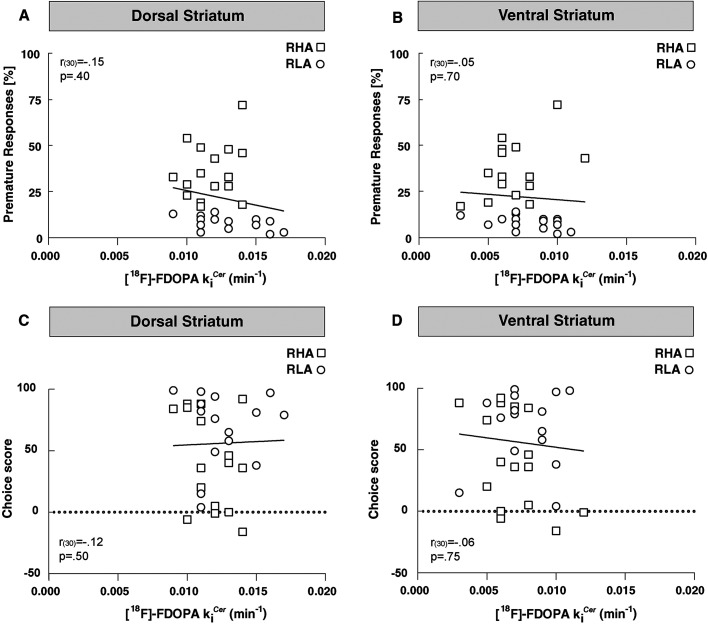
Relationships between striatal [^18^F]-FDOPA *k_i_*^cer^, impulsive action, and RDM in RHAs (□) and RLAs (○) at baseline. ***A***, ***B***, There was no correlation between [^18^F]-FDOPA *k_i_*^cer^ and premature responding in either the DST or VST. ***C***, ***D***, No correlation existed between [^18^F]-FDOPA *k_i_*^cer^ and choice score in either the DST or VST.

At baseline, there was no correlation between premature responding and [^18^F]-FDOPA *k_i_*^cer^ in either the DST (*r*_(30) _= −0.15; *p* = 0.40; Fig. 3*A*) or the VST (*r*_(30) _= −0.05; *p* = 0.70; Fig. 3*B*), indicating that striatal [^18^F]-FDOPA *k_i_*^cer^ does not predict impulsive action. Similarly, no correlation existed between the choice score and the baseline [^18^F]-FDOPA k*_i_*^cer^ in any striatal subdivision (*DST: r*_(30) _= −0.12, *p* = 0.50; *VST*: *r*_(30) _= −0.06, *p* = 0.75; Fig. 3*C,D*), indicating that striatal [^18^F]-FDOPA *k_i_*^cer^ is also not predictive of RDM.

### Intravenous cocaine self-administration

**Figure 4.**
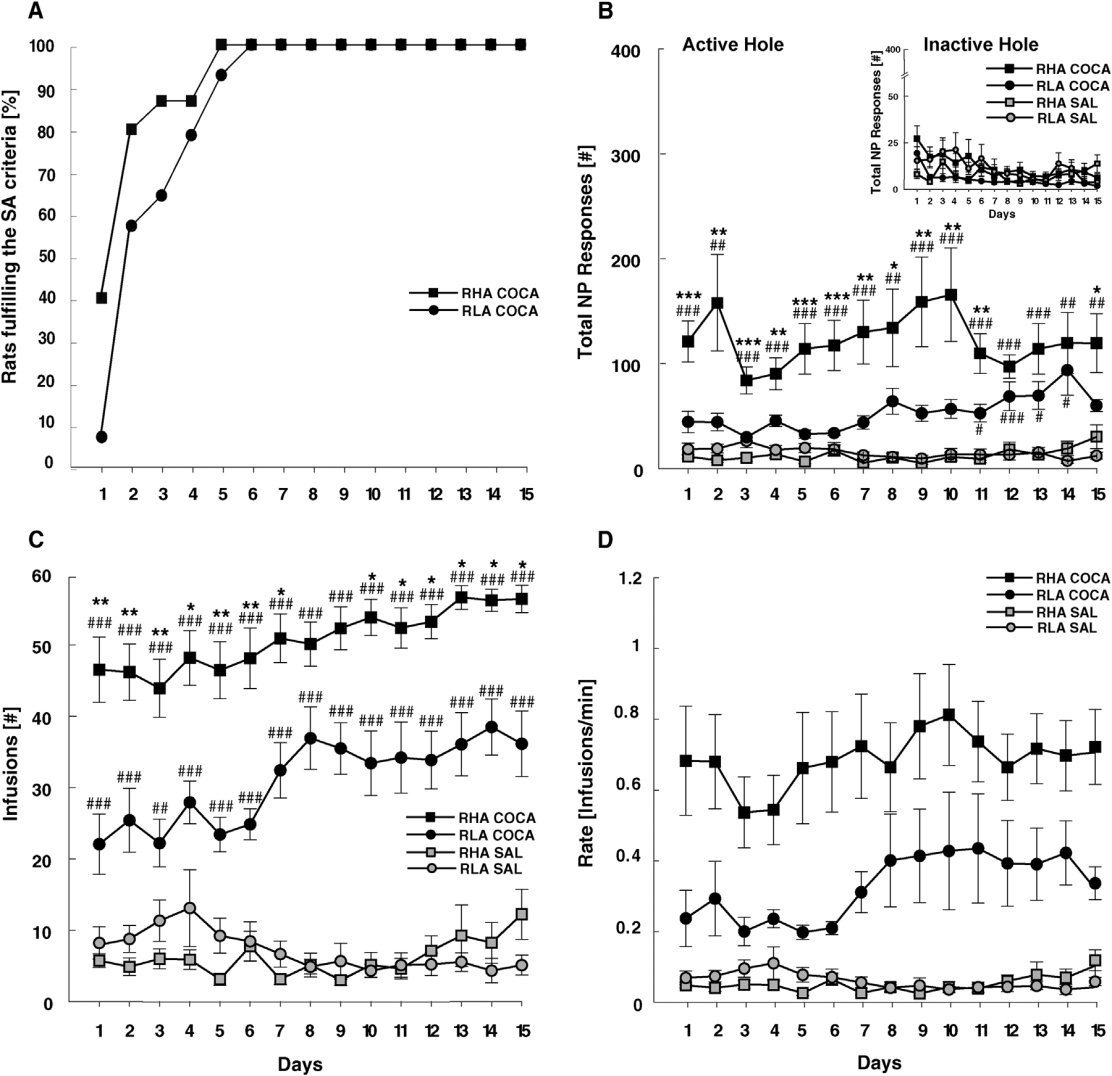
Comparison of cocaine and saline SA between RHA and RLA rats. ***A***, Cumulative percentage of rats meeting SA acquisition criteria (i.e., >15 infusions with 70% discrimination of the active vs inactive hole over 2 consecutive days). Only cocaine-exposed animals fulfilled the SA acquisition criteria, with RHAs acquiring cocaine SA significantly faster than RLAs. ***B***, Total nose-poke (NP) responses in the active hole were significantly higher in cocaine- than in saline-exposed rats and in RHAs compared with RLAs. The inserted image shows the responses in the inactive hole, which were similar between groups. ***C***, The number of infusions was significantly higher in cocaine-exposed rats than saline-exposed rats and in RHAs compared with RLAs. ***D***, The rate of cocaine infusions was significantly higher in RHA than that in RLA rats throughout the 15 d of SA. Data appears as mean ± SEM. Significantly different at *p < 0.05, **p < 0.01 in cocaine-exposed RHAs compared with cocaine-exposed RLAs, and ^##^*p* < 0.01, ^###^*p* < 0.001 between cocaine-exposed RHAs or RLAs relative to their corresponding saline control.

Figure 4 shows the acquisition and maintenance of cocaine and saline SA in RHA and RLA rats. SA acquisition (i.e., >15 infusions with 70% discrimination of the active hole over 2 consecutive days) occurred only in cocaine-exposed rats. In these rats, cocaine SA acquisition was faster in RHAs than that in RLAs (*χ*^2^ = 4.3; df = 1; *p* > 0.05; Fig. 4*A*), with 80% of the RHAs reaching the acquisition criteria on day 2, while the same proportion of RLAs reached those criteria by day 5.

A mixed factorial ANOVA on the number of nose-pokes in the active versus inactive hole (Fig. 4*B*) showed a main effect of treatment (*F*_(1,42) _= 5.05; *p* < 0.001; *ηp*^2^ = 0.34), line (*F*_(1,42) _= 5.05; *p* < 0.05; *ηp*^2^ = 0.11), hole (*F*_(1,588) _= 37.6; *p* < 0.001; *ηp*^2^ = 0.47), and treatment × line × hole interaction (*F*_(1,588) _= 5.14; *p* < 0.05; *ηp*^2^ = 0.11) with no effect of session (*F*_(1,588) _= 0.5; *p* = 0.93; *ηp*^2^ = 0.01). Post hoc comparisons further confirmed that only cocaine-exposed rats showed clear discrimination of the active versus inactive hole (RHAs, *p* < 0.001; RLAs, *p* < 0.001). Furthermore, cocaine-exposed RHAs performed statistically a higher number of nose-poke responses in the active hole than cocaine-exposed RLAs (*p* < 0.05). The number of infusions was also conditional to treatment (*F*_(1,42) _= 406.7; *p* < 0.001; *ηp*^2^ = 0.91) and rat's line (*F*_(1,42) _= 5.6; *p* < 0.05; *ηp*^2^ = 0.12), with RHAs self-injecting more cocaine than RLAs along the training (treatment × line × session: *F*_(14,588) _= 2.8; *p* = 0.006; *ηp*^2^ = 0.06; post hoc comparisons in RHA-Coca vs RLA-Coca, *p* < 0.05 except for days 8 and 9). Maintenance of cocaine SA (i.e., ≤15% variation in the number of infusions over 3 consecutive days) was also different between rat lines, with RHAs reaching the criteria of stable cocaine infusions on day 3, while RLAs reached the criteria on day 9 (Fig. 4*C*). Besides differences in cocaine taking, RHAs and RLAs also differed in how fast they self-injected cocaine (Fig. 4*D*). A mixed factorial ANOVA on the rate of cocaine infusion showed a main effect of treatment (*F*_(1,42) _= 31.3; *p* < 0.001; *ηp*^2^ = 0.43), line (*F*_(1,42) _= 4.7; *p* < 0.05; *ηp*^2^ = 0.10) and a treatment × line interaction (*F*_(1,42) _= 5.22; *p* < 0.05; *ηp*^2^ = 0.11), with no effect of session (*F*_(14,32) _= 0.80; *p* = 0.67; *ηp*^2^ = 0.01). Post hoc comparisons confirmed that cocaine-exposed RHAs were faster than cocaine-exposed RLAs to self-inject the drug (*p* < 0.05).

### Predictive value of impulsivity measures and DA synthesis on cocaine SA

**Figure 5.**
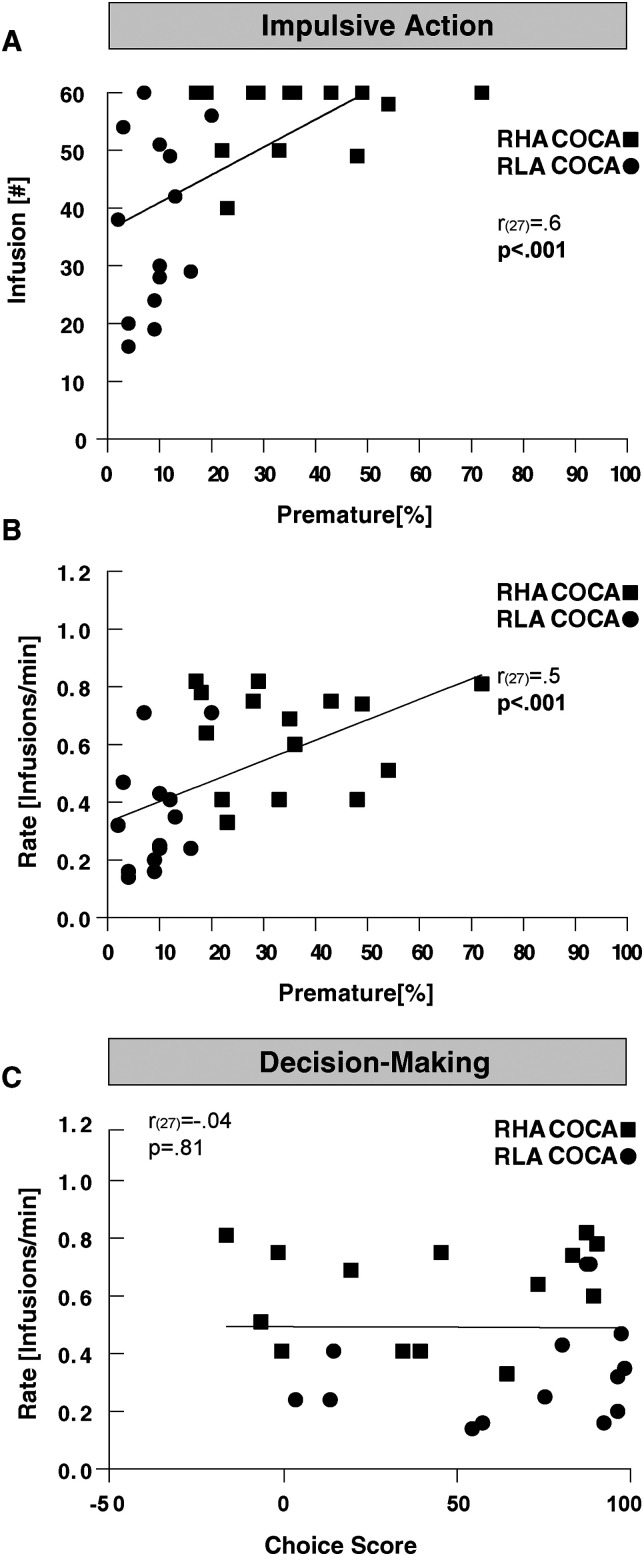
Relationship between impulsive action, RDM, and cocaine SA in RHA (▪) and RLA rats (●). Premature responding was significantly correlated with (***A***) the number of cocaine infusions earned and (***B***) the rate of cocaine infusions. ***C***, No correlation was found between the choice score and the rate of cocaine infusions.

There was a positive correlation between premature responding and the total number of infusions earned (*r*_(27) _= 0.6; *p* < 0.001; Fig. 5*A*). This correlation remained significant even after the removal of the rat with the highest percentage of premature responses (*r*_(26) _= 0.6; *p* = 0.0011). However, this correlation was potentially biased due to a ceiling effect resulting from the capped limit of 60 cocaine infusions allowed. Consequently, further analyses were conducted with the rate of cocaine infusions as a measure of cocaine taking. We observed a positive correlation between premature responding and the rate of cocaine infusions, indicating that the higher the premature responses, the faster the animals self-injected cocaine (*r*_(27) _= 0.5; *p* < 0.001; Fig. 5*B*). This correlation remained significant even after removal of the rat with the highest percentage of premature responses (*r*_(26) _= 0.5; *p* = 0.0025). No correlation was observed between premature responding and the total number of nose-poke responses in the active hole (*r*_(27) _= 0.3; *p* = 0.1). On the other hand, when evaluating the predictive value of the choice score, no correlation was found with the rate of cocaine infusions (*r*_(27) _= −0.04; *p* = 0.81; Fig. 5*C*) or the total exploration of the active hole (*r*_(27) _= 0.03; *p* = 0.8). These results indicated that impulsive action but not RDM predicts the propensity to cocaine taking.

**Figure 6.**
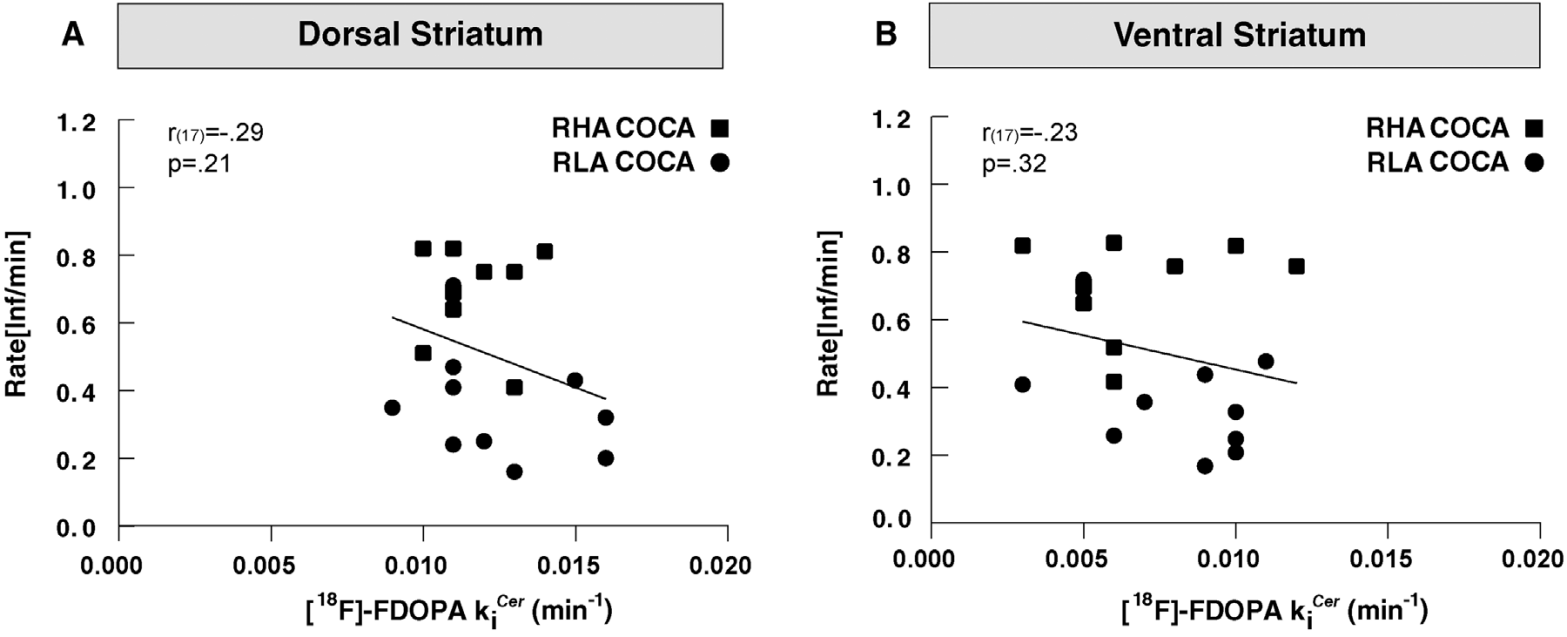
Relationship between [^18^F]-FDOPA *k_i_*^cer^ and cocaine SA in RHA (▪) and RLA rats (●). [^18^F]-FDOPA *k_i_*^cer^ in neither (***A***) DST nor (***B***) VST predicted the rate of cocaine infusions.

No significant correlation appeared between [^18^F]-FDOPA *k_i_*^cer^ and the rate of cocaine infusions (DST: *r*_(17) _= −0.29, *p* = 0.21; VST: *r*_(17) _= −0.23, *p* = 0.32; Fig. 6*A*,*B*), suggesting that DA synthesis capacity might not be a predictor of cocaine taking.

### Effect of repeated cocaine consumption on impulsive action and RDM

**Figure 7.**
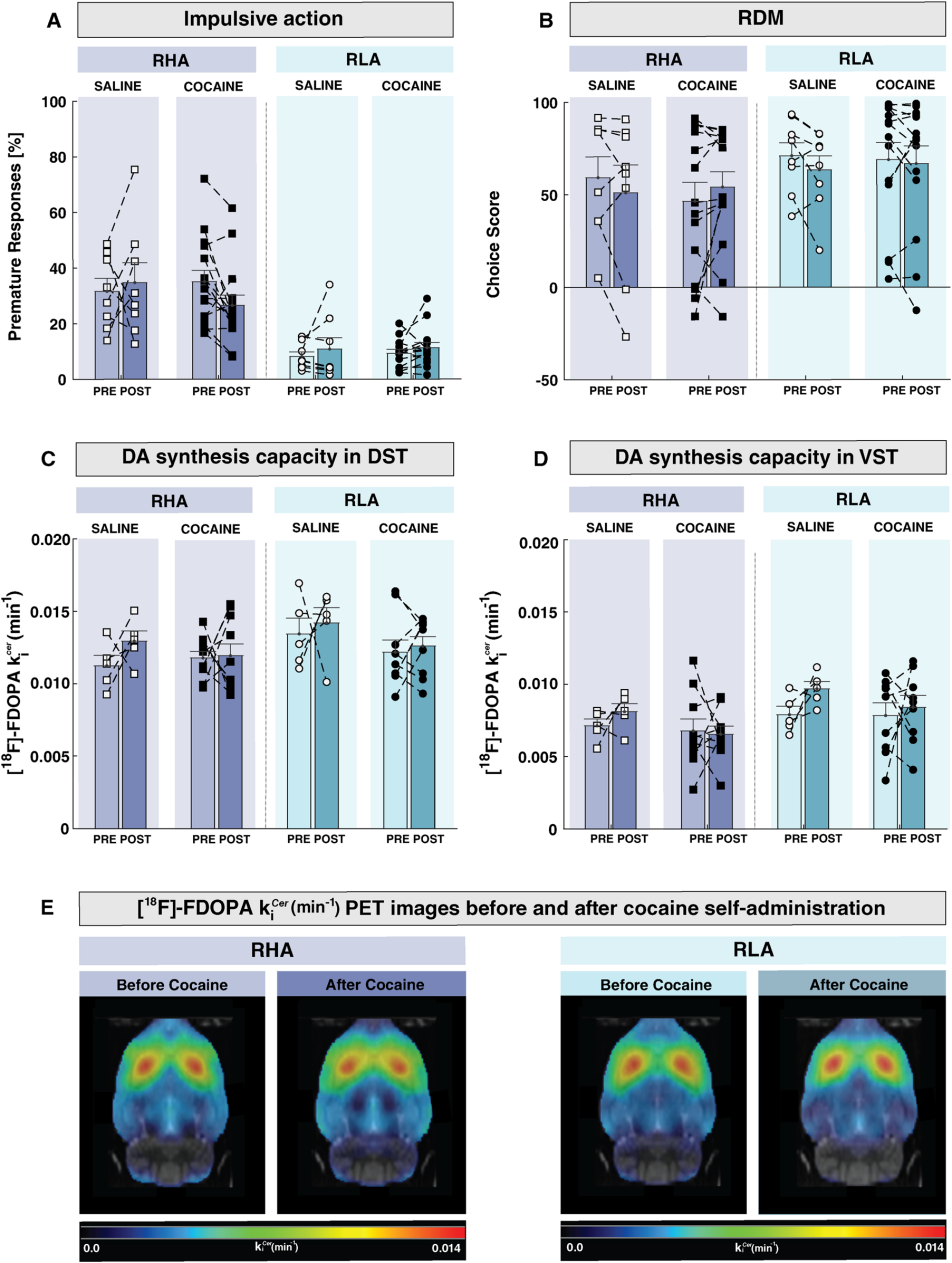
Effect of cocaine SA on impulsive action, RDM, and [^18^F]-FDOPA *k_i_*^cer^ in RHAs and RLAs. Cocaine SA did not alter (***A***) premature responding or (***B***) choice score in RHA or RLA rats. [^18^F]-FDOPA *k_i_*^cer^, as an index of DA synthesis capacity, was not altered following cocaine SA in either (***C***) DST or (***D***) VST, suggesting that repeated cocaine intake does not alter DA synthesis. ***E***, Averaged parametric maps of [^18^F]-FDOPA *k_i_*^cer^ in RHAs and RLAs before and after cocaine SA. Data are presented as mean ± SEM.

Figure 7, *A* and *B*, shows the effect of prior cocaine or saline SA on rGT behaviors. Neither saline nor cocaine SA affected the percentage of premature responses in RHA or RLA rats (treatment: *F*_(1,41) _= 0.22, *p* = 0.64, *ηp*^2^ = 0.005; treatment × line: *F*_(1,41) _= 0.82, *p* = 0.37, *ηp*^2^ = 0.02). Similarly, there was no effect of time (*F*_(1,41) _= 0.48; *p* = 0.49; *ηp*^2^ = 0.012) or time × line × treatment interaction (*F*_(1,41)_ = 3.6; *p* = 0.064; *ηp*^2^ = 0.08). These results indicate that baseline impulsive action is not significantly altered after cocaine or saline SA. Similarly, a mixed factorial ANOVA showed no effects of treatment (*F*_(1,41)_ = 0.48; *p* = 0.49; *ηp*^2^ = 0.12) or a treatment × line interaction (*F*_(1,41) _= 1.29; *p* = 2.6; *ηp*^2^ = 0.03) on choice score. Further, there was neither an effect of time (*F*_(1,41) _= 3.89; *p* = 0.06; *ηp*^2^ = 0.08) nor a time × line × treatment interaction (*F*_(1,41) _= 0.001; *p* = 0.97; *ηp*^2^ = 0) on choice score, indicating that cocaine or saline SA in both rat lines did not significantly alter RDM when compared with baseline. Finally, no changes in the number of trials occurred as a consequence of the treatment (*F*_(1,41) _= 0.3; *p* = 0.87; *ηp*^2^ = 0.001), treatment × line (*F*_(1,41) _= 0.43; *p* = 0.51; *ηp*^2^ = 0.01), time (*F*_(1,41) _= 2.06; *p* = 0.16; *ηp*^2^ = 0.04), or time × line × treatment interaction (*F*_(1,41) _= 0.58; *p* = 0.45; *ηp*^2^ = 0.01).

### Effect of repeated cocaine SA on striatal DA synthesis capacity

When compared with baseline measures, [^18^F]-FDOPA *k_i_*^cer^ in DST (Fig. 7*C*) did not significantly change after saline or cocaine SA in any rat line (treatment: *F*_(1,25) _= 2.7, *p* = 0.11, *ηp*^2^ = 0.09; treatment × line: *F*_(1,25) _= 1.4, *p* = 0.24, *ηp*^2^ = 0.05; time × line × treatment interaction: *F*_(1,25) _= 0.20, *p* = 0.65, *ηp*^2^ = 0.008). Similarly, an analysis of [^18^F]-FDOPA *k_i_*^cer^ in VST (Fig. 7*D*) showed no significant effect of treatment (*F*_(1,25) _= 3.76; *p* = 0.06; *ηp*^2^ = 0.13), treatment × line (*F*_(1,25) _= 0.01; *p* = 0.91; *ηp*^2^ = 0), time (*F*_(1,25) _= 0.06; *p* = 0.80; *ηp*^2^ = 0.002) or time × line × treatment interaction (*F*_(1,25) _= 0.0002; *p* = 0.99; *ηp*^2^ = 0). These results indicate that repeated cocaine taking does not alter baseline DA synthesis capacity in the striatum of RHA or RLA rats (Fig. 7*E*).

## Discussion

To our knowledge, this is the first longitudinal study in rats investigating the relationship between striatal DA synthesis, impulsive behaviors, and vulnerability to cocaine use. We replicated previous findings showing that RHA rats display higher impulsive action, less optimal decision-making, and higher cocaine taking than RLA rats (Dimiziani et al., 2019; Arrondeau et al., 2023; Bellés et al., 2023). Importantly, we extended those findings by showing that impulsive action and RDM are unlikely related to variations in DA synthesis. Additionally, our study shows that chronic cocaine use does not affect impulsive action or RDM, nor decreased DA synthesis, even though similar cocaine exposure has proven sufficient to blunt striatal DA release (Urueña-Mendez et al., 2023). Altogether, our study indicates that alterations in DA synthesis might not be the mechanism behind heightened-evoked DA release linked to impulsivity, nor the mechanism underlying dopaminergic tolerance to psychostimulants.

Considering that previous studies associated impulsive behaviors with heightened DA release (Bellés et al., 2021) and elevated TH mRNA in the midbrain (Tournier et al., 2013), it is somewhat unexpected that our study found no evidence of altered DA synthesis in impulsive rats. However, a recent study (Nago-Iwashita et al., 2023) showed that TH overexpression does not necessarily result in elevated DA levels, which suggests that additional regulatory mechanisms other than elevated TH expression underlie heightened DA release in impulsive subjects. Our findings of preserved DA synthesis in impulsive rats are also consistent with recent neuroimaging studies in humans, where DA synthesis was independent of trait impulsivity (van den Bosch et al., 2023) and RDM (Skandali et al., 2022), despite the relationship between impulsivity and elevated DA release (Buckholtz et al., 2010; Oswald et al., 2015). Our findings are also consistent with microdialysis studies in RHA rats (Giorgi et al., 2003; Lecca et al., 2004) or rats classified as high-impulsive (Zeeb et al., 2016), where basal-extrasynaptic or tonic DA levels were comparable to those of nonimpulsive rats. These observations suggest that the heightened evoked DA release linked to impulsive action and RDM is unlikely to stem from an overall elevation in DA synthesis or heightened tonic dopaminergic activity but may more likely arise from an enhanced reactivity of DA neurons to stimulation. Although no studies have directly measured DA cell firing at baseline and in response to stimuli (i.e., rewards or associated cues) in impulsive individuals, the hypothesis that impulsivity might result from altered phasic rather than tonic DA firing has received some indirect support. For instance, optogenetic stimulation of DA neurons in the ventral tegmental area increases premature responding (Flores-Dourojeanni et al., 2021; Suri et al., 2023) and leads to compulsive reward seeking (Seiler et al., 2022). This suggests that impulsive animals might exhibit a heightened reactivity of DA cells to stimulation, which may render them more vulnerable to the rewarding effects of drugs. However, future studies evaluating DA reactivity to rewards and associated cues in animals exhibiting different levels of impulsive behaviors will help to understand whether heightened phasic DA firing underlies impulsivity and the risk of cocaine abuse.

The uncoupling between normal DA synthesis and hyper-reactive DA release suggested here is unclear but might result from different mechanisms, including alterations in midbrain D_2_autoreceptor (D_2_AutoR)-inhibitory control of DA neurotransmission. D_2_AutoR negatively controls DA release through mechanisms beyond DA synthesis. For instance, D_2_autoR decreases dopaminergic cell excitability by activating potassium rectifying channels, inhibits DA release by blocking calcium influx, or facilitates DA reuptake by increasing the expression of the DA transporter (Ford, 2014). Hence, a reduction in D2AutoR, as reported in high-impulsive humans (Buckholtz et al., 2010) and rats (Besson et al., 2013; Bellés et al., 2021), could lead to heightened-evoked DA release through any of these mechanisms. Another potential mechanism that might account for an uncoupling between normal DA synthesis and heightened DA release is a deficit in top-down cortical control of DA cell firing. Lesioning or pharmacological inactivation of the medial prefrontal cortex (mPFC) has been shown to elevate impulsive action (Chudasama et al., 2003; Agnoli et al., 2013; Feja and Koch, 2014), RDM (Zeeb et al., 2015; van Holstein and Floresco, 2020), and phasic DA cell firing in the midbrain (Tan et al., 2014; Jo and Mizumori, 2016). Interestingly, in impulsive RHA rats, there is evidence of reduced glutamatergic transmission in the mPFC (Klein et al., 2014) and reduced mPFC volume (Río-Álamos et al., 2019) compared with low-impulsive RLA rats, which could potentially explain their increased impulsivity and hyper-reactive DA function. Furthermore, reduced cortical activity has been linked to higher RDM (Lv et al., 2021) and drug abuse in humans (Volkow et al., 1993) and may thus also contribute to a higher vulnerability for cocaine abuse. However, additional experimental evidence is needed to substantiate this hypothesis.

Our results demonstrate for the first time, using a within-subject design, that chronic cocaine exposure does not alter DA synthesis, regardless of baseline levels of impulsivity or the facet of impulsivity assessed. This finding suggests that a reduction in DA synthesis is probably not the mechanism responsible for the attenuation of evoked DA release that is observed in cocaine abusers (Martinez et al., 2007; Volkow et al., 2014) and impulsive animals with a history of cocaine use (Urueña-Mendez et al., 2023). Despite substantial evidence of attenuated DA release in drug abusers (Nutt et al., 2015), studies measuring DA synthesis in this population have been limited, and their results are inconclusive. Consistent with our findings, a study in polydrug users observed no alterations in DA synthesis relative to healthy controls (Tai et al., 2011). Conversely, another study found marked reductions in DA synthesis in cocaine abusers (Wu et al., 1997). Similar contrasts were reported for other drugs, such as cannabis (Bloomfield et al., 2014a) and smoked nicotine (Bloomfield et al., 2014b; Rademacher et al., 2016). Likewise, two studies in rats had previously measured striatal L-DOPA accumulation after passive cocaine injections, finding either a decrease (Trulson and Ulissey, 1987) or no alterations (Baumann et al., 1993) in exposed animals versus controls. However, it was not clear whether these results were generalizable to self-administered cocaine, a more human-relevant drug-taking pattern, nor whether the effects varied in impulsive animals. By using within-subjects measures of DA synthesis and a cocaine SA protocol able to blunt striatal DA release in impulsive rats (Urueña-Mendez et al., 2023), our study indicates that reduced DA synthesis might not underlie cocaine-induced DA tolerance, irrespective of baseline impulsivity. Thus, alternative mechanisms might be considered to understand the decreased DA release observed in drug abusers and impulsive rats after chronic cocaine use. For instance, reduced sensitivity of the DA transporter (Mateo et al., 2005; Ferris et al., 2011), increased sensitivity or expression of D_2_AutoR (King et al., 2004; Stefański et al., 2007; Clare et al., 2021), or reduced vesicular release (Little et al., 2003; Narendran et al., 2015) have also been observed after chronic cocaine use and could independently lead to reduced capacity of DA release.

Besides exhibiting blunted DA release, cocaine abusers exhibit heightened impulsive behaviors (Kjome et al., 2010; Voon et al., 2014), raising the question of whether impulsivity represents a pre-existing risk factor or the consequence of cocaine use. Our data suggest that impulsive action, but not RDM, predicts cocaine taking, further supporting the view that impulsive action is a stronger predictor than RDM of the rewarding effects of cocaine (Arrondeau et al., 2023) and thus represents an endophenotype of vulnerability to the drug. However, despite continuous research, some debate still exists on the effects of cocaine on specific impulsivity facets. For instance, as shown in our data and consistent with several studies (Dalley et al., 2005; Ferland and Winstanley, 2017; Abbott et al., 2022; Urueña-Mendez et al., 2023), chronic cocaine exposure did not significantly alter impulsive action (but see also Dalley et al., 2007; Winstanley et al., 2009; Caprioli et al., 2013). In contrast, the evidence regarding RDM has been less consistent. While previous studies showed impairments in RDM either in risky rats (Ferland and Winstanley, 2017) or in nonrisky rats (Cocker et al., 2019), our results show that chronic cocaine use does not alter RDM, irrespective of baseline levels. Although the reason for this discrepancy is unclear, they might reflect differences in the dose of cocaine use. For instance, we used a dose of cocaine that was less than half the dose used in the previous studies. While this dose is sufficient to induce a high response rate in Roman rats (Dimiziani et al., 2019) and trigger DA neuroadaptations under chronic use (Urueña-Mendez et al., 2023), we cannot exclude the possibility that RDM is affected only at higher doses of cocaine.

### Study limitations

While RHA rats showed less optimal decision-making than RLA rats, such RDM levels might not be representative of pathological states. Thus, although we observed no alterations in DA synthesis in relation to RDM, DA dynamics might differ in animals with more risky profiles, as suggested by human studies, where heightened RDM in pathological gamblers was linked to increased DA synthesis (van Holst et al., 2018). It is important to note that while our study did not find significant differences in DA synthesis between RHA and RLA rats, nor did it establish a predictive relationship between DA synthesis and cocaine taking, it is possible that the PET sample size may have constrained the statistical power to detect subtle effects. However, the generally small effect sizes observed suggest that, if any, the association between DA synthesis and cocaine taking is likely to be moderate. Another potential limitation of our study is the use of only one temporal window to evaluate the effects of cocaine on DA synthesis. While cocaine could have transient effects on DA synthesis immediately after discontinuation, such potential effects would be unlikely to underlie the blunted DA release induced by cocaine, which is observed even 12 d after cocaine discontinuation (Urueña-Mendez et al., 2023). It is also important to acknowledge that while brain [^18^F]-FDOPA uptake is conventionally interpreted as an indicator of DA synthesis and storage, it also encompasses additional factors such as the release of synthesized [^18^F]dopamine and its subsequent turnover (Sossi et al., 2003; Kumakura and Cumming, 2009). However, without a direct measure of brain [^18^F]-FDOPA metabolites, we cannot entirely disentangle a possible contribution of these additional factors to our measurement. Finally, our study included only males, while the mechanisms controlling DA release may vary in females (Zachry et al., 2021), who are also reported as more vulnerable to cocaine intake than males (Knouse and Briand, 2021). Thus, further studies should consider evaluating sex differences in DA synthesis and its role in impulsivity and cocaine abuse.

In conclusion, our findings revealed that, unlike DA release (Bellés et al., 2021), striatal DA synthesis might not predict impulsivity or the propensity to cocaine consumption, nor is it altered after chronic cocaine exposure. This suggests that changes in DA synthesis would not be the potential mechanism behind excessive DA release linked to impulsivity, nor the mechanism underlying DA tolerance to psychostimulants. This uncoupling between DA release and synthesis suggests the existence of additional regulatory mechanisms, such as altered DA firing to stimulation, that could contribute to heightened impulsivity and vulnerability to cocaine abuse.

## Synthesis

Reviewing Editor: Michael Michaelides, NIDA-NIH Decisions are customarily a result of the Reviewing Editor and the peer reviewers coming together and discussing their recommendations until a consensus is reached. When revisions are invited, a fact-based synthesis statement explaining their decision and outlining what is needed to prepare a revision will be listed below. The following reviewer(s) agreed to reveal their identity: David Zald.

Reviewer 1

This preclinical study with Roman High and Low Avoidance rat lines provides evidence for a relationship between impulsivity and cocaine self-administration, but fails to provide evidence for hypothesized associations between dopamine synthesis capacity and impulsive action/premature responding or risky decision making, or an impact of chronic cocaine use on dopamine synthesis. Overall, the rationale for the study is clear, and the methods appear reasonable. Although the primary findings related to dopamine synthesis are all negative, it is important for such negative results to be published, as they can help narrow hypotheses about the specific properties of dopamine that are related to behavior and addiction.

There are, nevertheless, a few features of the study that require attention.

A couple of issues in terms of describing limitations are needed. First, some discussion of the level of statistical power in the study is needed. Many of the nonsignificant results have low enough effect sizes that it is reasonably safe accepting the null hypotheses, but other results the relatively modest sample size could provide some caution for conclusions. For instance, the difference in [18F]-FDOPA kicer between the two rat lines (Line: F(1,30) =2.38,265 p=.13, ηp2=.06) or the correlation appeared between [18F]-FDOPA kicer and the rate of cocaine infusions(DST: r(17)=-.29, p=.21; VST: r(17)=-.23 p=.32, Fig. 6. A-B). Certainly, these negative findings indicate that if there is a relation it is likely to be modest, but the strength of current conclusions have to be considered relative to the statistical power of the tests. Second, some discussion of methodological issues related to FDOPA measurement is needed, as such measurements involve more than just synthesis, since the synthesized dopamine is also released and turned over which impacts measurement.

There are also some issues related to coverage of the existing literature.

First, in the introduction there is a clear misstatement. The authors state that "humans with high trait impulsivity exhibit reductions in striatal dopamine (DA) D2/3 receptors (D2/3R) (Buckholtz et al., 2010) as those observed in cocaine abusers (Volkow et al., 1993)." But Buckholtz does not show a baseline reduction in D2/D3 receptor availability in the striatum (their report of baseline lowered binding potential is focused on the midbrain (with effects in the striatum relating to psychostimulant induced DA release). It may also be noted that although the Volkow et al. 1993 is a seminal paper, there is at least one far more recent meta-analysis that provides stronger evidence in support of the lowered D2 receptors (Ashok, A. H., Mizuno, Y., Volkow, N. D., & Howes, O. D. (2017). Association of stimulant use with dopaminergic alterations in users of cocaine, amphetamine, or methamphetamine: a systematic review and meta-analysis. JAMA psychiatry, 74(5), 511-519).

In the review of the existing literature on chronic cocaine use and DA synthesis, the authors may wish to consider prior preclinical work by Trulson and Ulisse 1987.

Additionally, in the human literature, the authors may wish to consider inclusion of Smith, Christopher T., et al. "Modulation of impulsivity and reward sensitivity in intertemporal choice by striatal and midbrain dopamine synthesis in healthy adults." Journal of neurophysiology 115.3 (2016): 1146-1156. (although perhaps this is not being mentioned because it represents a different type of impulsivity than premature responding).

Reviewer 2

The authors report the results of a technically accomplished study to investigate the relationship between striatal dopamine synthesis and (1) impulsive action; (2) risky decision making, and (3) the propensity to self administer intravenous cocaine. Prior research has implicated various mechanisms linked to striatal dopamine neurotransmission to the underlying propensity of humans and non-human animals to self-administer psychostimulant drugs, but surprisingly few studies have specifically looked at dopamine synthesis as an explanatory mechanistic process. The experiment appears to have been carefully carried out with appropriate statistical analyses and discussion. Although mostly null findings are reported, the study nevertheless holds significant value to the scientific community.

This is a high quality piece of research but null findings inevitably invite the concern that some of procedures may have been insufficiently sensitive to detect genuine effects on dopamine synthesis or, relatedly, the way in which these procedures were implemented may have had an unintended bearing on the results. Firstly, can the authors be sure that the [18F]DOPA signal would decrease in the striatum if dopamine synthesis was inhibited by a pharmacological agent? A positive control experiment would be a reassuring way of validating the dependent PET variable. Secondly, the authors acknowledge that cocaine exposure levels may have been insufficient to alter dopamine synthesis but this point needs to more robustly defended and refuted with explicit evidence from previous studies that unequivocally rule out the possibility of cocaine insufficiency in the present study. This points relates to the unit cocaine dose, session duration, and the total period of cocaine self-administration. Thirdly, what effect does 12 hour fasting have on dopamine synthesis rate? Could it be that fasting makes it less likely to detect changes in dopamine synthesis? Fourthly, is there any evidence to show that altered dopamine synthesis rapidly recovers when cocaine is discontinued? This may be relevant as the second PET scan was effected no sooner than 3-days after the last cocaine self-administration session. Fifthly, could the null findings be explained by testing male rats only? Lastly, it would be worth considering whether the present null findings obtained in inbred RHA and RLA rats would generalize to other rodent (mouse and rat) strains, including outbred strains.

These six putative explanations should each be considered on their merits and either retained or discounted so the reader can form a balanced view of the validity of the null findings reported.

The correlations shown in Figs.5A and 5B look to be driven by one extremely impulsive animal. Do the significant correlations survive if this animal is removed from the correlational analyses?

As NET is also a primary target of cocaine it might be worth noting whether NE synthesis is affected by the discontinuation of cocaine self-administration.

## Author Response

Dear Reviewing Editor Michael Michaelides,

We appreciate the reviewers' prompt and insightful review of our manuscript, and we are grateful for the opportunity to submit a revised version that addresses all of the reviewers' comments comprehensively.

In this revised manuscript, we have specifically addressed the reviewers' concerns by incorporating an additional correlational analysis, additional references from the literature, and text changes that clarify our arguments. We believe that these revisions have significantly improved the overall quality of the manuscript, and we hope that it will deemed for publication in eNeuro.

Yours sincerely,

Prof. Nathalie GINOVART

-

We thank the reviewers for their thoughtful and comprehensible evaluation of the manuscript. We have carefully considered their suggestions and have incorporated changes throughout the paper accordingly.

Reviewer 1.

Q1: A couple of issues in terms of describing limitations are needed. First, some discussion of the level of statistical power in the study is needed. Many of the nonsignificant results have low enough effect sizes that it is reasonably safe accepting the null hypotheses, but other results the relatively modest sample size could provide some caution for conclusions. For instance, the difference in [18F]-FDOPA kicer between the two rat lines (Line: F(1,30) =2.38 p=.13, ηp2=.06) or the correlation appeared between [18F]-FDOPA kicer and the rate of cocaine infusions (DST: r(17)=-.29, p=.21; VST: r(17)=-.23 p=.32, Fig. 6. A-B). Certainly, these negative findings indicate that if there is a relation it is likely to be modest, but the strength of current conclusions have to be considered relative to the statistical power of the tests.

Author's Reply: Thank you for your thoughtful comment regarding the statistical power of certain measures. We agree that this is an important consideration, and we have addressed it by adding a remark within the limitations section of the manuscript and by writing more nuanced conclusions where pertinent (as highlighted in bold below):

Study Limitations (PDF page 22, lines 474-480)

"It is important to note that while our study did not find significant differences in DA synthesis between RHA and RLA rats, nor did it establish a predictive relationship between DA synthesis and cocaine-taking, it is possible that the PET sample size may have constrained the statistical power to detect subtle effects. However, the generally small effect sizes observed suggest that, if any, the association between DA synthesis and cocaine-taking is likely to be moderate."

Abstract (PDF page 1, lines 13-15)

"Our results revealed that baseline striatal levels of DA synthesis did not significantly predict impulsive action, RDM, or a greater propensity to cocaine self-administration (SA) in impulsive animals."

Abstract (PDF page1, lines 17-21)

"These findings indicate that the hyperresponsive DA system associated with impulsivity and a propensity for cocaine consumption, along with the reduction in this hyperresponsive DA state in impulsive animals with a history of cocaine use, might not be mediated by dynamic changes in DA synthesis."

Results (PDF page 15, lines 328-330)

"No significant correlation appeared between [18F]-FDOPA kicer and the rate of cocaine infusions..., suggesting that DA synthesis capacity might not be a predictor of cocaine-taking."

Discussion (PDF page 18, lines 363-364)

"Importantly, we extended those findings by showing that impulsive action and RDM are unlikely related to variations in DA synthesis."

Discussion (PDF page18, lines 367-369)

"Altogether, our study indicates that alterations in DA synthesis might not be the mechanism behind heightened-evoked DA release linked to impulsivity."

Discussion (PDF page19, lines 384-385)

"These observations suggest that the heightened evoked DA release linked to impulsive action and RDM is unlikely to stem from an overall elevation in DA synthesis."

Discussion (PDF page 21, lines 440-441)

"Our study indicates that reduced DA synthesis might not underlie cocaine-induced DA tolerance, irrespective of baseline impulsivity."

Conclusion (PDF page 23, lines 496-497)

"Striatal DA synthesis might not predict impulsivity or the propensity to cocaine consumption."

Conclusion (PDF page 23, lines 498-499)

"This suggests that changes in DA synthesis would not be the potential mechanism behind excessive DA release linked to impulsivity..."

Figure 2. Legend (PDF Page 35, lines 775-776)

D) [18F]-FDOPA kicer did not significantly differ between RHA and RLA rats in either DST or VST

Q2: Second, some discussion of methodological issues related to FDOPA measurement is needed, as such measurements involve more than just synthesis, since the synthesized dopamine is also released and turned over which impacts measurement.

Author's Reply: We thank you for your important suggestion. We have carefully considered your feedback and have incorporated a new section in the Limitations section of the manuscript as follows:

Study Limitations (PDF page 23, lines 484-490)

"It is also important to acknowledge that while brain [18F]-FDOPA uptake is conventionally interpreted as an indicator of DA synthesis and storage, it also encompasses additional factors such as the release of synthesized [18F]dopamine and its subsequent turnover (Kumakura and Cumming, 2009; Sossi et al., 2003). However, without a direct measure of brain [18F]-FDOPA metabolites, we cannot entirely disentangle a possible contribution of these additional factors to our measurement"

Q3: There are also some issues related to coverage of the existing literature. First, in the introduction, there is a clear misstatement. The authors state that "humans with high trait impulsivity exhibit reductions in striatal dopamine (DA) D2/3 receptors (D2/3R) (Buckholtz et al., 2010) as those observed in cocaine abusers (Volkow et al., 1993)." But Buckholtz does not show a baseline reduction in D2/D3 receptor availability in the striatum (their report of baseline lowered binding potential is focused on the midbrain (with effects in the striatum relating to psychostimulant induced DA release.

Author's Reply: We are grateful for the careful examination of our manuscript and for bringing to our attention the inaccuracy of the citation. We have carefully corrected the citation as follows.

Introduction (PDF page 3, lines 45-47)

"For instance, humans with high trait impulsivity exhibit reductions in striatal dopamine (DA) D2/3 receptors (D2/3R) (Caravaggio et al., 2016)..."

Q4: It may also be noted that although the Volkow et al. 1993 is a seminal paper, there is at least one far more recent meta-analysis that provides stronger evidence in support of the lowered D2 receptors (Ashok, A. H., Mizuno, Y., Volkow, N. D., & Howes, O. D. (2017). Association of stimulant use with dopaminergic alterations in users of cocaine, amphetamine, or methamphetamine: a systematic review and meta-analysis. JAMA psychiatry, 74(5), 511-519).

Author's Reply: We appreciate your suggestion to cite Ashok et al. 2017. This meta-analysis is now referenced in the revised manuscript as follows.

Introduction (PDF page 3, lines 45-48)

"For instance, humans with high trait impulsivity exhibit reductions in striatal dopamine (DA) D2/3 receptors (D2/3R) (Caravaggio et al., 2016) as those observed in cocaine abusers (Ashok et al., 2017; Volkow et al., 1993)."

Q5: In the review of the existing literature on chronic cocaine use and DA synthesis, the authors may wish to consider prior preclinical work by Trulson and Ulisse 1987.

Author's Reply: Thank you for your thoughtful suggestion of adding the work by Trulson and Ulisse; it certainly helps us provide a more accurate representation of the current state of knowledge on the topic. We have made the necessary modifications to add the suggested reference as follows.

Discussion (PDF page 21, lines 433-438)

"Likewise, two studies in rats had previously measured striatal LDOPA accumulation after passive cocaine injections, finding either a decrease (Trulson and Ulissey, 1987) or no alterations (Baumann et al., 1993) in exposed animals vs controls. However, it was unclear whether these results were generalizable to self-administered cocaine, a more human-relevant drug-taking pattern, nor whether the effects varied in impulsive animals."

Q5: Additionally, in the human literature, the authors may wish to consider the inclusion of Smith, Christopher T. et al., "Modulation of impulsivity and reward sensitivity in intertemporal choice by striatal and midbrain dopamine synthesis in healthy adults." Journal of Neurophysiology 115.3 (2016): 1146-1156. (although perhaps this is not being mentioned because it represents a different type of impulsivity than premature responding).

Author's Reply: We appreciate your valuable suggestion of including the work by Smith et al. within the human literature cited. However, within the scope of our manuscript, we tried to maintain the focus on premature responses and risk-related decision-making.

Reviewer 2.

Q1: This is a high-quality piece of research, but null findings inevitably invite the concern that some of the procedures may have been insufficiently sensitive to detect genuine effects on dopamine synthesis or, relatedly, the way in which these procedures were implemented may have had an unintended bearing on the results. Firstly, can the authors be sure that the [18F]DOPA signal would decrease in the striatum if dopamine synthesis was inhibited by a pharmacological agent? A positive control experiment would be a reassuring way of validating the dependent PET variable.

Author's Reply: Thank you for your insightful comment regarding the reliability of our PET imaging results. We understand your concern about the possibility of false negatives and the need for a positive control experiment to validate the dependent variable. However, there is evidence in the literature that [18F]-FDOPA uptake responds to pharmacological interventions. For instance, chronic exposure to high doses of amphetamine, known for inducing sustained depletion of striatal DA levels, has been demonstrated to result in long-lasting, yet reversible, reductions in striatal [18F]-FDOPA uptake in monkeys (Melega et al., 1997). Acute treatment with haloperidol, which acutely disinhibits DA synthesis via D2 autoreceptor blockade, has been shown to increase striatal [18F]-FDOPA uptake (Vernaleken et al., 2006). Conversely, prolonged D2-autoreceptor blockade through subchronic haloperidol treatment, leading to "depolarization block" and silencing of midbrain DA neurons, decreases [18F]-FDOPA uptake (Gründer et al., 2003). We thus believe that these pharmacological studies provide evidence supporting the sensitivity of the [18F]-FDOPA signal to detect changes in DA synthesis

Q2: Secondly, the authors acknowledge that cocaine exposure levels may have been insufficient to alter dopamine synthesis, but this point needs to be more robustly defended and refuted with explicit evidence from previous studies that unequivocally rule out the possibility of cocaine insufficiency in the present study. This point relates to the unit cocaine dose, session duration, and the total period of cocaine self-administration.

Author's Reply: We appreciate the reviewers' careful consideration of our work, and we would like to respectfully highlight that there might be a possible misunderstanding of our statement here. Our intention was not to acknowledge that cocaine exposure levels might have been insufficient to influence DA synthesis but rather that they might have been insufficient to affect RDM.

In several instances within the manuscript (page 18, lines 366-367; page 21, lines 439), we highlighted that the cocaine self-administration protocol employed in our study did not result in a reduction of DA synthesis, even though we previously showed that similar cocaine exposure levels led to a decrease in striatal DA release (Urueña-Mendez et al., 2023). Our intent was to underscore that, despite utilizing a cocaine exposure protocol previously proven to effectively decrease striatal DA release in impulsive rats, we did not observe a concurrent reduction in DA synthesis in our present investigation. This observation implies that, at the administered dose, chronic cocaine may not directly influence DA synthesis despite its ability to induce changes in DA release.

On page 22 (lines 463-467), which we believe is the part of the manuscript the reviewer is referring to, we mentioned that our cocaine self-administration protocol did not produce any impact on RDM, in contrast to the only two studies conducted in the field. It is in an effort to explain this discrepancy that we acknowledge the possibility that our administered dose of cocaine, lower than that used in the two aforementioned studies, might have been insufficient to influence RDM.

We hope that this clarification will address any potential misunderstandings or doubts the reviewer may have had regarding the adequacy of our study.

Q3: Thirdly, what effect does 12-hour fasting have on dopamine synthesis rate? Could it be that fasting makes it less likely to detect changes in dopamine synthesis?

Author's Reply: Fasting before a [18F]-FDOPA PET scan is recommended to enhance the detection of changes in DA synthesis, particularly in the brain. Indeed, fasting enhances the relative bioavailability of [18F]-FDOPA in the brain and contributes to a higher signal-to-noise ratio in the images, which is essential for obtaining high-quality results. Additionally, fasting helps standardize the conditions of the PET scan, ensuring consistency and comparability of results across subjects. Consequently, overnight fasting is commonly incorporated into standard neuroimaging protocols using [18F]-FDOPA, even in clinical populations. Such fasting is unlikely to decrease the likelihood of detecting changes in DA synthesis. Indeed, multiple studies conducted across diverse clinical populations have shown variations in [18F]-FDOPA uptake in individuals who underwent overnight fasting before the PET scan. For example, increased striatal DA synthesis has been documented in patients with schizophrenia (Gefvert et al., 2003; Kumakura et al., 2007) and in those at ultra-high risk for psychosis (Egerton et al., 2013; Howes et al., 2011). Conversely, decreased striatal DA synthesis has been observed in patients with a cannabis use disorder (Bloomfield et al., 2014). A recent study even reported higher DA synthesis in individuals with 22q11.2 deletion and lower synthesis in those with 22q11.2 duplication compared to healthy controls (Rogdaki et al., 2023). While these examples are not exhaustive of all studies using overnight fasting, they collectively indicate that alterations in [18F]-FDOPA uptake can be observed despite overnight fasting, underscoring that fasting does not preclude the likelihood of observing changes in DA synthesis.

Q4: Fourthly, is there any evidence to show that altered dopamine synthesis rapidly recovers when cocaine is discontinued? This may be relevant as the second PET scan was effected no sooner than 3-days after the last cocaine self-administration session.

Author's Reply: Thank you for your insightful comment regarding the timing of DA synthesis changes. It is hypothetically possible that cocaine affects DA synthesis immediately after discontinuation or up to 2 days, but as far as we know, such evidence is not available for chronic cocaine use. However, even in the case that DA synthesis was reduced transiently shortly after cocaine SA, it would be unlikely that DA synthesis was the mechanism underlying blunted DA release induced by cocaine because, as demonstrated in our previous study, such blunted DA release is observed even 12 days after cocaine discontinuation.

Based on your comment, we have rephrased the limitation of the temporal window of measure as follows:

Study Limitations (PDF pages 22-23, lines 480-484)

Another potential limitation of our study is the use of only one temporal window to evaluate the effects of cocaine on DA synthesis. While cocaine could have transient effects on DA synthesis immediately after discontinuation, such potential effects would be unlikely to underlie the blunted DA release induced by cocaine, which is observed even 12 days after cocaine discontinuation (Urueña-Mendez et al., 2023).

Q5: Fifthly, could the null findings be explained by testing male rats only?

Author's Reply: We appreciate your observation regarding the potential influence of having testing males only. While there are human studies on both male and female participants showing no correlation between DA synthesis and goal-directed behaviors (Voon et al., 2020), for example, suggesting that null findings in our study might not necessarily be due to the use of male rats only, we cannot exclude that our results necessarily apply when considering female rats.

We tried to address this limitation in the manuscript on the page 23, lines 490-494, by stating that "our study included only males, while the mechanisms controlling DA release may vary in females (Zachry et al., 2021), who are also reported as more vulnerable to cocaine intake than males (Knouse and Briand, 2021). Thus, further studies should consider evaluating sex differences in DA synthesis and its role in impulsivity and cocaine abuse."

Q6: Lastly, it would be worth considering whether the present null findings obtained in inbred RHA and RLA rats would generalize to other rodent (mouse and rat) strains, including outbred strains.

Author's Reply: We agree that it is important to consider the generalizability of our findings to other rodent strains. While our study was conducted using outbred RHA and RLA rats, previous results using those lines have also been observed in other rat strains. For instance, using Roman rats, we have previously shown a relation between impulsive action and cocaine intake (Arrondeau et al., 2023), impulsive action and reduced striatal D2/3R (Bellés et al., 2021), or a relation between impulsive action and RDM (Bellés et al., 2023). Similar results have also been reported in other rat strains, such as Lister hooded rats (Besson et al., 2013; Dalley et al., 2007), or Sprague Dawley (Barrus et al., 2015), and even most interestingly, they have also been observed in humans, as mentioned in the manuscript. In light of these converging lines of evidence, we believe that our results hold broad applicability to other rodent strains and have a potential impact on human research.

Q7: The correlations shown in Figs.5A and 5B look to be driven by one extremely impulsive animal. Do the significant correlations survive if this animal is removed from the correlational analyses?

Author's Reply: We appreciate your input regarding the potential influence of that animal on the correlations depicted in Figs.5A and 5B. To address this concern, we re-ran the correlational analyses after excluding that highly impulsive animal, and the correlations remained statistically significant. This has been explicitly stated in the paper as follows:

Results (PDF page 15, lines 313-314)

There was a positive correlation between premature responding and the total number of infusions earned (r(27)=.6 p<.001, Fig. 5A). This correlation remained significant even after the removal of the rat with the highest percentage of premature responses (r(26)=.6 p= .0011).

Results (PDF page 15, lines 320-321)

We observed a positive correlation between premature responding and the rate of cocaine infusions, indicating that the higher the premature responses, the faster the animals self-injected cocaine (r(27)=.5 p<.001, Fig. 5B). This correlation remained significant even after removal of the rat with the highest percentage of premature responses (r(26)=.5 p=.0025).

Q8: As NET is also a primary target of cocaine, it might be worth noting whether NE synthesis is affected by the discontinuation of cocaine self-administration.

Author's Reply: As mentioned, cocaine also impacts the NE system, and it is recognized that [18F]-FDOPA, in norepinephrine-rich regions such as the cortex, serves as a combined indicator of both DA and NE synthesis (Cumming, 2009). However, [18F]-FDOPA uptake in the striatum is considered to reflect more specifically DA function. In the striatum, NE terminals and transporters are relatively sparse compared to DA terminals (Ranjbar-Slamloo and Fazlali, 2020). Additionally, DA release in the striatum is 13 times greater than NE release (Ihalainen et al., 1999). Further, there is evidence that [18F]-FDOPA uptake is markedly higher in the striatum than in the cortex (Nagano et al., 2000), which is consistent with our observation that cortical [18F]-FDOPA uptake was extremely low. Given these considerations, we believe that [18F]-FDOPA uptake in the striatum predominantly reflects DA, rather than NE, synthesis and that our data indicate a lack of alterations in DA synthesis rather than effects on NE synthesis after cocaine discontinuation.-

References

Arrondeau C, Urueña-méndez G, Bellés L, Marchessaux F, Goutaudier R (2023) Motor impulsivity but not risk-related impulsive choice is associated to drug intake and drug-primed relapse 1-14.

Ashok AH, Mizuno Y, Volkow ND, Howes OD (2017) Association of stimulant use with dopaminergic alterations in users of cocaine, amphetamine, or methamphetamine a systematic review and meta-analysis. JAMA Psychiatry 74:511-519.

Barrus MM, Hosking JG, Zeeb FD, Tremblay M, Winstanley CA (2015) Disadvantageous decision-making on a rodent gambling task is associated with increased motor impulsivity in a population of male rats. J Psychiatry Neurosci 40:108-117.

Bellés L, Arrondeau C, Urueña-Méndez G, Ginovart N (2023) Concurrent measures of impulsive action and choice are partially related and differentially modulated by dopamine D1- and D2-like receptors in a rat model of impulsivity. Pharmacol Biochem Behav 222.

Bellés L, Dimiziani A, Herrmann FR, Ginovart N (2021) Early environmental enrichment and impoverishment differentially affect addiction-related behavioral traits, cocaine-taking, and dopamine D2/3 receptor signaling in a rat model of vulnerability to drug abuse. Psychopharmacology (Berl) 238:3543-3557.

Besson M, Pelloux Y, Dilleen R, Theobald DE, Lyon A, Belin-Rauscent A, Robbins TW, Dalley JW, Everitt BJ, Belin D (2013) Cocaine modulation of frontostriatal expression of Zif268, D2, and 5-HT2c receptors in high and low impulsive rats. Neuropsychopharmacology 38:1963-1973.

Bloomfield MAP, Morgan CJA, Egerton A, Kapur S, Curran HV, Howes OD (2014) Dopaminergic function in cannabis users and its relationship to cannabis-induced psychotic symptoms. Biol Psychiatry 75:470-478.

Cumming P (2009) Imaging Dopamine. New York: Cambrigge University Press.

Dalley JW, Fryer TD, Brichard L, Robinson ESJ, Theobald DEH, Lääne K, Peña Y, Murphy ER, Shah Y, Probst K, Abakumova I, Aigbirhio FI, Richards HK, Hong Y, Baron JC, Everitt BJ, Robbins TW (2007) Nucleus accumbens D2/3 receptors predict trait impulsivity and cocaine reinforcement. Science (80- ) 315:1267-1270.

Egerton A, Chaddock CA, Winton-Brown TT, Bloomfield MAP, Bhattacharyya S, Allen P, McGuire PK, Howes OD (2013) Presynaptic striatal dopamine dysfunction in people at ultra-high risk for psychosis: Findings in a second cohort. Biol Psychiatry 74:106-112.

Gefvert O, Lindström LH, Waters N, Waters S, Carlsson A, Tedroff J (2003) Different corticostriatal patterns of L-DOPA utilization in patients with untreated schizophrenia and patients treated with classical antipsychotics or clozapine. Scand J Psychol 44:289-292.

Gründer G, Vernaleken I, Müller MJ, Davids E, Heydari N, Buchholz HG, Bartenstein P, Munk OL, Stoeter P, Wong DF, Gjedde A, Cumming P (2003) Subchronic haloperidol downregulates dopamine synthesis capacity in the brain of schizophrenic patients in vivo. Neuropsychopharmacology 28:787-794.

Howes OD, Bose SK, Turkheimer F, Valli I, Egerton A, Valmaggia LR, Murray RM, McGuire P (2011) Dopamine synthesis capacity before onset of psychosis: A prospective [ 18F]-DOPA PET imaging study. Am J Psychiatry 168:1311-1317.

Ihalainen JA, Riekkinen P, Feenstra MGP (1999) Comparison of dopamine and noradrenaline release in mouse prefrontal cortex, striatum and hippocampus using microdialysis. Neurosci Lett 277:71-74.

Kumakura Y, Cumming P (2009) PET studies of cerebral levodopa metabolism: A review of clinical findings and modeling approaches. Neuroscientist 15:635-650.

Kumakura Y, Cumming P, Vernaleken I, Buchholz HG, Siessmeier T, Heinz A, Kienast T, Bartenstein P, Gründer G (2007) Elevated [18F]fluorodopamine turnover in brain of patients with schizophrenia: An [18F]fluorodopa/positron emission tomography study. J Neurosci 27:8080-8087.

Melega WP, Raleigh MJ, Stout DB, Huang SC, Phelps ME (1997) Ethological and 6-[18F]fluoro-L-DOPA-PET profiles of long-term vulnerability to chronic amphetamine. Behav Brain Res 84:259-268.

Nagano AS, Ito K, Kato T, Arahata Y, Kachi T, Hatano K, Kawasumi Y, Nakamura A, Yamada T, Abe Y, Ishigaki T (2000) Extrastriatal mean regional uptake of fluorine-18-FDOPA in the normal aged brain - An approach using MRI-aided spatial normalization. Neuroimage 11:760-766.

Ranjbar-Slamloo Y, Fazlali Z (2020) Dopamine and Noradrenaline in the Brain; Overlapping or Dissociate Functions? Front Mol Neurosci 12:1-8.

Rogdaki M, Devroye C, Ciampoli M, Veronese M, Ashok AH, McCutcheon RA, Jauhar S, Bonoldi I, Gudbrandsen M, Daly E, van Amelsvoort T, Van Den Bree M, Owen MJ, Turkheimer F, Papaleo F, Howes OD (2023) Striatal dopaminergic alterations in individuals with copy number variants at the 22q11.2 genetic locus and their implications for psychosis risk: a [18F]-DOPA PET study. Mol Psychiatry 28:1995-2006.

Sossi V, Holden JE, De la Fuente-Fernandez R, Ruth TJ, Stoessl AJ (2003) Effect of dopamine loss and the metabolite 3-O-methyl-[18F]fluoro-dopa on the relation between the 18F-fluorodopa tissue input uptake rate constant Kocc and the [18F]fluorodopa plasma input uptake rate constant Ki. J Cereb Blood Flow Metab 23:301-309.

Vernaleken I, Kumakura Y, Cumming P, Buchholz HG, Siessmeier T, Stoeter P, Müller MJ, Bartenstein P, Gründer G (2006) Modulation of [18F]fluorodopa (FDOPA) kinetics in the brain of healthy volunteers after acute haloperidol challenge. Neuroimage 30:1332-1339.

Volkow ND, Fowler JS, Wang GJ, Hitzemann R, Logan J, Schlyer JD, Dewey SL, Wolf A (1993) Decreased dopamine D2 receptor availability is associated with reduced frontal metabolism in cocaine abusers.pdf.

Voon V, Joutsa J, Majuri J, Baek K, Nord CL, Arponen E, Forsback S, Kaasinen V (2020) The neurochemical substrates of habitual and goal-directed control. Transl Psychiatry 10.
